# Supplementary material for: Study Protocol for a Hospital-to-Home Transitional Care for Older Adults Hospitalized with Chronic Obstructive Pulmonary Disease in South Korea: A Randomized Controlled Trial
Source: Int J Environ Res Public Health. 2023 Aug 2;20(15):6507. doi: 10.3390/ijerph20156507 (PMC10418954; doi:10.3390/ijerph20156507)
Supplement: Supplementary file 1 [file ijerph-20-06507-s001.zip › ijerph-2345060-supplementary.pdf]

International Journal of
*Environmental Research and Public Health*

MDPI

## Supplementary Material

**Table. S1** Study measurements

| Timepoint | | Training | Eligibility Screening | Enrollment ($t_1$) | Allocation | Post-Allocation | | | Follow-up | |
|---|---|---|---|---|---|---|---|---|---|---|
| | | | | within 72 hours of hospitalization | | In the hospital | Within 48 hours before discharge | Within 48 hours of discharge | 1m ($t_2$) | 3m ($t_3$) |
| **Enrolment :** | | | | | | | | | | |
| Training of CRC, Multidisciplinary team | | X | | | | | | | | |
| Eligibility Screen by CRC | | | X | | | | | | | |
| Invitation to Participate | | | | X | | | | | | |
| Validation and Informed Consent | | | | X | | | | | | |
| Participant Randomization | | | | | X | | | | | |
| **Intervention :** | | | | | | | | | | |
| Usual Care | | | | | | X | | | X | X |
| Discharged Patient Health Management Program | Education | | | | | X | X | X | X | |
| | Linking social welfare | | | | | X | | | X | X |
| | Home Visiting | | | | | | | X | X | |
| | Telephone counseling | | | | | | | | x x x x | |
| **Measures :** | | | | | | | | | | |
| Modified Medical Research Council (mMRC) Dyspnea Scale | | | | X | | | | | X | X |
| COPD Assessment Test (CAT) | | | | X | | | | | X | X |
| Demonstration of inhaler technique | | | | X | | | | | X | X |

| | | | |
|---|---|---|---|
| Test of the Adherence to Inhalers (TAI) | X | X | X |
| Hospitalization and emergency room visits | X | X | X |
| Exacerbation History | X | X | X |
| Demographic questions | X | X | X |
| Smoking | X | X | X |
| Prescreening Korean Dementia Screening Questionnaire (KDSQ-P) | X | X | X |
| Hospital anxiety and depression scale (HADS) | X | X | X |
| Self-efficacy (SE) scale | X | X | X |
| Necessity for Social Welfare Service | X | X | X |
| Instrumental Activities of Daily Living (IADL) / Activities of Daily Living (ADL) | X | X | X |
| Patient Experience Assessment (PEA) | X | | |
| Partners at Care Transitions Measure (PACT-M1) | X | | |
| Partners at Care Transitions Measure (PACT-M2) | | X | X |
